# Supplementary material for: Deletion of wfs1 Impairs Oligodendrocyte Precursor Cells Dorsal Distribution and Myelination Through the wfs1-hmgcs1 Axis in Zebrafish
Source: Biology (Basel). 2026 Aug 16;15(16):1402. doi: 10.3390/biology15161402 (PMC13510151; doi:10.3390/biology15161402)
Supplement: Supplementary file 1 [file biology-15-01402-s001.zip › File S1-Transcriptome sequencing data and differentially expressed genes (DEGs) in wfs1-- zebrafish at 5 dpf/2.KEGG/wfs1KOvsWT/UP/src/dre00240.html]

dre00240


- dre:100330864

- Up regulated genes

100330864(6.66489299727258)

- dre:550560

- Up regulated genes

550560\_1(1.24185328918673)

- dre:100005763

- Up regulated genes

100005763(1.20717146570446)
- dre:558466

- Up regulated genes

558466\_1(2.16978955072169)

- dre:100005763

- Up regulated genes

100005763(1.20717146570446)
- dre:558466

- Up regulated genes

558466\_1(2.16978955072169)

- dre:492517

- Up regulated genes

492517(1.99607785946957)

- dre:100330864

- Up regulated genes

100330864(6.66489299727258)

- dre:393744

- Up regulated genes

393744\_1(1.33052857104334)

- dre:492517

- Up regulated genes

492517(1.99607785946957)

- dre:103910413

- Up regulated genes

103910413\_1(2.52090153714663)

- dre:103910413

- Up regulated genes

103910413\_1(2.52090153714663)

Close
